# FBXW8 suppresses PDCoV proliferation via the NPD52-dependent autophagic degradation of a viral nucleocapsid protein

**DOI:** 10.3389/fimmu.2024.1457255

**Published:** 2024-11-18

**Authors:** Likai Ji, Liying Zhou, Ying Wang, Shixing Yang, Yuwei Liu, Xiaochun Wang, Quan Shen, Chenglin Zhou, Juan Xu, Wen Zhang

**Affiliations:** ^1^ Institute of Critical Care Medicine, The Affiliated People’s Hospital, Jiangsu University, Zhenjiang, China; ^2^ School of Medicine, Jiangsu University, Zhenjiang, China; ^3^ Clinical Laboratory Center, The Affiliated Taizhou People’s Hospital of Nanjing Medical University, Taizhou, China

**Keywords:** FBXW8, PDCoV, N protein, NDP52, selective autophagy

## Abstract

Porcine deltacoronavirus (PDCoV), a newly discovered intestinal coronavirus, has rapidly spread among pigs worldwide and has shown the potential for cross-species infection. However, the interaction mechanism between PDCoV and the host’s antiviral response is still poorly understood. In this study, an E3 ubiquitin ligase FBXW8 was explored on PDCoV proliferation. Our findings demonstrate that PDCoV infection increases the expression of FBXW8 through p65-mediated activation of its promoter. We also discovered that FBXW8 suppresses PDCoV replication by directly targeting and inducing the degradation of the PDCoV-encoded nucleocapsid (N) protein. Interestingly, FBXW8 catalyzes the K48-linked polyubiquitination of the PDCoV N protein at a unique lysine-rich region (KR). Furthermore, we observed that the FBXW8-ubiquitinated PDCoV N protein interacts with NDP52, a cargo receptor, leading to autophagic degradation instead of proteasomal degradation. In summary, these findings reveal FBXW8 as a novel host antiviral factor involved in PDCoV infection. It mediates the NDP52-dependent autophagic degradation of the PDCoV N protein. These results provide new insights and a potential target for host defenses against PDCoV.

## 1 Introduction

Porcine deltacoronavirus (PDCoV) is an enveloped, positive-sense RNA virus that causes severe disease in swine. PDCoV is classified into the *Deltacoronavirus* genus, which is newly identified in the family *Coronaviridae*. PDCoV was first detected in Hong Kong in 2012 and was the cause of a subsequent epidemic outbreak infecting pigs worldwide (1, 2). PDCoV infection causes the reduction of intestinal goblet cells and intercellular adhesion protein (ZO1), resulting in the destruction of the intestinal mucosal barrier and acute diarrhea, dehydration, vomiting, and even death especially in newborn piglets (2–4). In addition to causing serious economic losses in infected pigs, PDCoV has also been found to infect cattle, chicken, mice, and humans (5–7). These accumulating lines of evidence indicate that PDCoV has the potential for cross-species transmission and zoonotic capability, highlighting its status as a potentially emerging virus that poses a threat to public health security.

The host’s innate immune system, depending on its pattern recognition receptors (PRRs) to recognize pathogen, is the first line of defense against invading viruses. Classically, signals from the innate immune response of antigen-presenting cells can also be relayed to the adaptive immune system, which results in the elimination of the viruses by prime naive CD4+ T cells (8). PDCoV-infected piglet and intestinal organoids induced weak expression of interferon and interferon-stimulated genes, but high expression of TNFα (4). However, PDCoV infection suppresses the expression of type I and type III IFNs, which are important antiviral and immunomodulatory factors (9, 10). As the most abundant viral protein following infection, the PDCoV N protein not only helps pack the viral genome of its offspring but also interferes with RIG-I’s ability to sensor viral dsRNA and mediate IRF7 degradation, thereby inhibiting type I IFN production. However, the biological process of PDCoV N protein accumulation and degradation regulate remains unknown in viral infected cells.

Ubiquitination is an important protein post-translational modification (PTM) for protein activation or degradation of substrate protein. Essential molecules for identifying and attaching to certain substrates are E3 ligases. In mammals, the Cullin (Cul1–7)–Ring ligase (CRL) complexes have been identified as the largest subfamily of the RING-type E3 ligase family, which are responsible for up to 20% of all ubiquitinated substrates in cells (11). The most well-characterized CRLs are SKP1-Cul-F-box (SCF) complexes, which bind substrates in a variety of F-box protein-dependent ways. F-box proteins are classified into three categories: FBXW, which has a WD40-repeat domain; FBXL, which has a leucine-rich-repeat domain; and FBXO, which has no recognizable domain or another kind of protein interaction domain. The SCF complexes change the substrate protein PTMs to affect the innate immunity, such as FBXO6, FBXO17, and FBXO3 (12–14). Moreover, several F-box proteins were also identified to associate with viral encoded proteins of coronavirus, such as SARS-CoV-2 and PDCoV (5, 15).

F-box and WD repeat domain containing 8 (FBXW8) is the only member of F-box proteins to bind to the N-terminus of Cul7. The interaction with Cul7 depends on the WD40 domain of FBXW8, not its F-box domain (16). Cul7 C-terminus binds Rbx1 or Rbx2 to recruit the ubiquitin charged E2 ubiquitin conjugating enzyme, forming a protein complex (CRL7FBXW8) (17). FBXW8 is responsible for recruiting the substrate protein that is subjected to specific PTMs. FBXW8 plays an irreplaceable role in regulating cell cycle progression and signal transduction via mediating the degradation of substrate proteins. β-TrCP1 mediates the expression of several cyclins to maintain the stability of cell cycle. FBXW8 binds to the phosphorylated β-TrCP1 to direct its proteasomal degradation via forming the Cul1-SKP1-FBXW8-Cul7 functional complex (18). In mammalian brain neurons, FBXW8 mediates the Golgi protein Grasp65 degradation to control Golgi and dendrite morphogenesis in neurons (19). Moreover, few other cellular proteins have been reported as CRL7FBXW8 substrates, including IRS-1 and HPK1 (20, 21). Our previous study identified that FBXW8 was an important interactor of the PDCoV N protein (5). However, the role of FBXW8 in virus infection is still not understood.

In the present study, E3 ligase FBXW8 was newly identified in response to PDCoV infection. Furthermore, we observed that FBXW8 exerts an inhibitory impact on PDCoV replication. Mechanically, FBXW8 promotes the K48-linked polyubiquitination of the PDCoV N protein following the NPD52-dependent degradation via the autophagy–lysosomal pathway.

## 2 Materials and methods

### 2.1 Cell culture and virus

HEK-293T, PK-15, and LLC-PK1 cells (ATCC) were maintained in Dulbecco’s modified Eagle’s medium (DMEM) supplemented with 10% fetal bovine serum (Sigma, USA) and maintained at 37°C in 5% CO_2_. The emerging PDCoV Shanghai strain was cultured and stored as described in our previously study (5, 22).

### 2.2 Plasmids

The full-length coding sequence (CD) of porcine or human FBXW8 was cloned into plasmid pcDNA3.1-Flag, pcDNA3.1-Myc, or pcDNA3.1-HA to generate tagged proteins. Several mutations (Mut1: 125–532, Mut2: 1–124, Mut3: 272–532, and Mut4: 1–271) of FBXW8 were cloned into plasmid pcDNA3.1-Flag and confirmed by sequencing, including amino acid region. Five autophagy receptors (p62/SASTM1, NDP52/CALCOCO2, OPTN, BNIP3L, and TOLLIP) were constructed into the pcDNA3.1-Flag plasmid, respectively. PDCoV-N and its truncated plasmids with different tag-proteins, HA-tagged ubiquitin (Ub), Ub-K48R, and Ub-K63R were used as described previously (22). The Flag-tagged PDCoV N without the lysine-rich region (KR) (Flag-PDCoV-NdKR) was cloned from the Flag-PDCoV-N plasmid with specifically designed primers and constructed into the pcDNA3.1-Flag plasmid. The pGL3-Basic vector, pRL-TK luciferase reporter plasmid, and Dual-Glo Luciferase Assay System were purchased from Promega. A 1,909 base pairs (bp) of the FBXW8 promoter sequence (designated P1) and its nine truncated promoters (designated P2–P9) were cloned into the pGL3-Basic vector. All primer information of the above expression plasmids is presented in **Supplementary Table 1**.

### 2.3 Coimmunoprecipitation assay

LLC-PK1 cells were uninfected or infected with PDCoV for 28 h. HEK-293T cells were co-transfected with specific plasmids for 28 h. These cells were lysed with ice-cold lysis buffer [50 mM Tris-HCl (pH 7.4), 150 mM NaCl, 1% NP-40, 10% glycerin, 0.1% SDS, and 2 mM Na_2_EDTA] containing protease inhibitor cocktail and phosphatase inhibitor cocktail. Later, the whole-cell lysates were centrifuged and incubated at 4°C with mouse anti-Flag or anti-HA affinity gel for 4 h (Beyotime, China), followed by rinsing three times with 1×Tris-buffered saline. Endogenous FXBW8 protein was immunoprecipitated from PDCoV uninfected or infected LLC-PK1 cell lysate using FBXW8 (A18122, ABclonal, China) or IgG (AC005, ABclonal, China) antibody and coupled to protein A/G beads (36403ES03; YEASEN, China). The mouse anti-PDCoV N polyclonal antibodies were prepared by our laboratory. Immunoblotting (IB) was then performed to analyze proteins with specific antibodies.

### 2.4 GST affinity isolation assay

We inserted full-length sequences of PDCoV N in pET28a-GST plasmid. The pCold-GST-NDP52 was kindly provided by Prof. Tongling Shan, Shanghai Veterinary Research Institute, Chinese Academy of Agricultural Sciences (SHVRI, CAAS). These genes were respectively expressed within the BL21(DE3) competent cells (C504-03; Vazyme Biotech). Protein interactions were examined with a GST protein interaction pulldown kit (21516; Thermo) by following specific protocols. Coomassie brilliant blue staining and Western blotting assay were performed for protein analysis after elution using reduced glutathione.

### 2.5 Luciferase reporter assay

In selected experiments, the plasmids were transfected into PK-15 or HEK-293T cells cultured within 24-well plates using Lipofectamine 6000 (Beyotime, China). The cells were lysed to measure their luciferase activities at 24 h post-transfection by adopting a Dual-Glo luciferase assay system (DL101; Vazyme Biotech Co., Ltd.). The Renilla luciferase was used as a reference to normalize the data.

### 2.6 Confocal immunofluorescence assay

PK-15 cells were co-transfected with specific plasmids. After 24-h transfection, cells were fixed by 4% paraformaldehyde (Beyotime, China) and then permeabilizated with 0.1% Triton X-100 (Beyotime, China) for 10 min under ambient temperature. After blocking with 5% bovine serum albumin (BSA) for 1 h, cells were incubated separately with primary antibody for 1 h. The cells were then rinsed thrice with PBS, followed by incubation with fluorescently labeled secondary antibody for another 1 h in the dark. The nuclei were subsequently treated with 4',6-diamidino-2-phenylindole (DAPI) for 5 min. Finally, the cells were observed with a confocal immunofluorescence microscope (Carl Zeiss, Jena, Germany).

### 2.7 RNA extraction and quantitative real-time PCR

Total RNA was extracted from cultured cells using Trizol reagent (Invitrogen) and was reverse-transcribed into cDNA using reverse transcriptase (TaKaRa, Japan). Quantitative real-time PCR (qRT-PCR) experiments were performed in triplicate. Relative mRNA expression levels were normalized to the expression level of GAPDH. All qRT-PCR experiments were performed using Low ROX SYBR Green PCR master mix (Vazyme, China) and an ABI 7300 Real-time PCR system. The primer sequences are presented in **Supplementary Table 1**.

### 2.8 Statistical analysis

Data from three independent experiments were expressed as means ± standard deviations. Significance was determined with two-tailed Student’s *t*-test to analyze the differences in multiple groups (≥3). *p*-values of <0.05 were considered statistically significant.

## 3 Results

### 3.1 PDCoV infection induces FBXW8 production via the transcription factor p65

**Figure 1:**
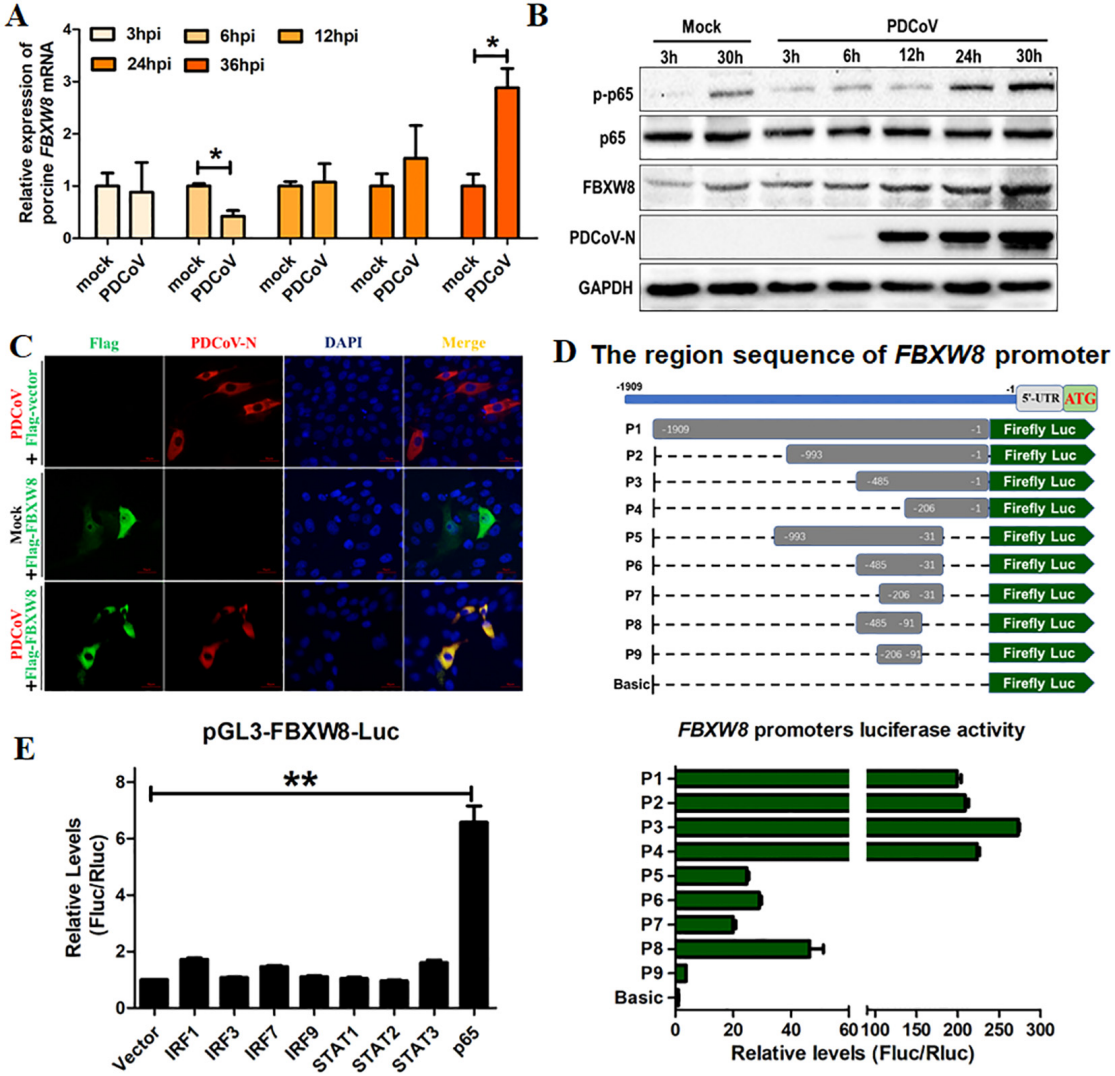
FBXW8 was upregulated by p65 and translocated to the cytoplasm in PDCoV-infected cells. **(A, B)** LLC-PK1 cells were infected with PDCoV (MOI of 1) for 3, 6, 12, 24, 30, or 36h and then collected for qRT-PCR detection **(A)** or Western blot analysis **(B)**. **(C)** Flag-tagged FBXW8 plasmids were transfected into PK15 cells with or without PDCoV infection. **(D)** HEK-293T cells were co-transfected with truncated FBXW8 promoter constructs (1909 to 1, P1 to P9) (up) and pRL-TK-luc. These cells were then analyzed for dual luciferase activity at 24 h post-transfection (down). **(E)** HEK-293T cells were transfected with the FBXW8 promoter-driven luciferase vector, pRL-TK-luc vector, and plasmids encoding Flag-tagged putative transcription factors (IRF1, IRF3, IRF7, IRF9, STAT1, STAT2, STAT3, or p65). Samples were collected at 24 h post-transfection and analyzed for dual luciferase activity. *p < 0.05; **p < 0.01.

F-box E3 ligase-mediated ubiquitination of substrate proteins plays an important role in the host’s antiviral process (23–25). In the present study, the FBXW8 expression in PDCoV-infected cells was examined to investigate its possible role in antiviral responses. LLC-PK1 cells were infected with PDCoV for different times and collected to analyze with qRT-PCR and Western blotting. The mRNA levels of FBXW8 were downregulated in PDCoV-infected cells at 6 hours post-infection (hpi), but significantly upregulated in PDCoV-infected cells at 36 hpi, compared to the levels in uninfected cells (**Figure 1A**). The expression of FBXW8 protein significantly increased in PDCoV-infected cells at 30 hpi, as demonstrated by Western blotting (**Figure 1B**). Moreover, FBXW8 was only expressed in the cytoplasm of PDCoV-infected cells, while it was also located in the nucleus of PDCoV-uninfected cells (**Figure 1C**). It indicated that PDCoV infection could regulate FBXW8 expression and translocation in cells.

To explore the mechanism of PDCoV-induced FBXW8 transcription, the promoter of FBXW8 was first analyzed. We amplified 1,909 bp of the FBXW8 promoter sequence and cloned it into a luciferase vector (pGL3-Basic) (named P1) (**Figure 1D**). A series of truncated promoters (designated P2–P9) were cloned into the luciferase vector, and their ability to direct luciferase expression in 293T cells was tested. The promoter deletion mutants containing nucleotides from −993 to −1 (P2, P3, and P4) induced the same luciferase expression as the full-length (P1) promoter (**Figure 1D**). The promoter deletion mutants containing nucleotides from −993 to −31 (P5, P6, and P7) and −485 to −91 (P8) induced the significantly lower luciferase expression than the full-length (P1) promoter, but significantly higher than the basic vector (**Figure 1D**). The promoter deletion mutants containing nucleotides from −206 to −91 (P9) could not significantly induce luciferase expression (**Figure 1D**). These results indicate that the region of the FBXW8 promoter at −485 to −207 bp was important for its transcription. Furthermore, we found that the FBXW8 promoter contains several transcription factor binding sites (TFBS, including STAT1, STAT2, IRF1, and NF-κB binding sites) with the JASPAR vertebrate database (http://jaspar.genereg.net/). To assess the effects of different transcription factors on the FBXW8 promoter in directing the expression of the gene, sequences encoding all the putative transcription factors were cloned into a mammalian expression vector and transfected with the FBXW8 promoter-driven luciferase vector to test their ability to direct luciferase expression in 293T cells. Cells overexpressing STAT1, STAT2, and IRF1/3/7/9 protein showed very low luciferase activity (**Figure 1E**). In contrast, cells overexpressing p65 (the subunit of the NK-κB complex) showed significantly increased luciferase expression from the FBXW8 promoter (**Figure 1E**). Consistent with the expression of FBXW8, p65 was also significantly upregulated in PDCoV-infected cells at 24 and 30 hpi (**Figure 1B**). Overall, these results indicate that PDCoV could promote the expression of FBXW8 by activating NF-κB and inducing p65 transfer into the nucleus.

### 3.2 FBXW8 suppresses the replication of PDCoV

**Figure 2:**
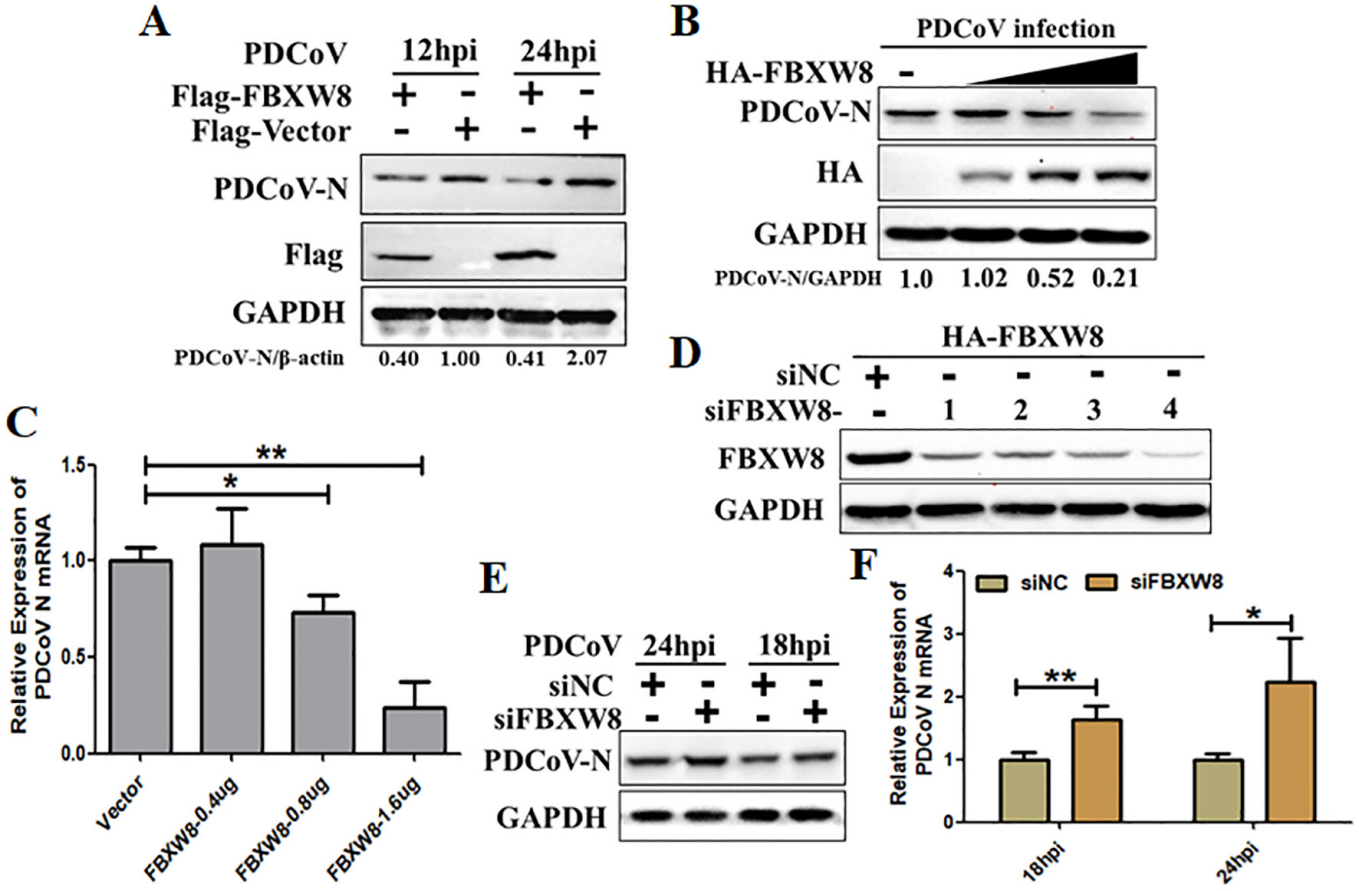
FBXW8 inhibits PDCoV proliferation in porcine cells. **(A)** The FBXW8 plasmids were overexpressed in PK15 cells and infected with 0.1 MOI of PDCoV for 12 and 24h. The cellular proteins were detected using Western blotting. **(B)** PK-15 cells were transfected with varying concentrations of the FBXW8 plasmids and subsequently infected with PDCoV (MOI = 0.1). Protein samples were collected for performing Western blot analysis. **(C)** The relative expression of PDCoV N mRNA was determined using qRT-PCR with the samples described in **(B)**. **(D)** The interference efficiency of four FBXW8 siRNAs (siFBXW8-1 to -4) were determined by Western blot analysis. **(E, F)** PK-15 cells were transfected with siFBXW8-4 and a negative control, and later infected with PDCoV (MOI = 0.05). The relative expression of PDCoV N protein or mRNA was analyzed using Western blot **(E)** or qRT-PCR **(F)** assay, respectively. *p < 0.05; **p < 0.01.

To study the function of FBXW8 in PDCoV infection, the study primarily evaluated whether FBXW8 influences PDCoV replication *in vitro*. The HA-tagged FBXW8 plasmids (HA-FBXW8) were transfected into LLC-PK1 cells for 24 h. Then, the cells were subjected to PDCoV infection (MOI = 0.1) and were collected for the indicated time points. Based on Western blotting, it was found that FBXW8 overexpression significantly suppressed PDCoV N protein expression at 12 and 24 hpi (**Figure 2A**). Furthermore, PDCoV N protein expression dose-dependently decreased with the increasing transfection dose of FBXW8 in PDCoV-infected PK15 cells (**Figure 2B**). Consistently, the PDCoV N mRNA expression presented significant downregulation in a dose-dependent manner (**Figure 2C**). To further explore the role of FBXW8 on PDCoV replication *in vitro*, four siRNAs targeting different locations of *FBXW8* exons were designed for synthesis. The results showed that all these siRNAs significantly downregulated the FBXW8 expression in PK-15 cells, especially siRNA-4 (**Figure 2D**). Hence, siRNA-4 was transfected into PK-15 cells and subsequently exposed to infection with PDCoV (MOI = 0.05) at 24 h post-transfection. Simultaneously, PDCoV N protein expression and PDCoV viral load were measured by Western blotting and qRT-PCR at 18 and 24 hpi. These results revealed that FBXW8 silencing significantly increased the PDCoV N protein and its mRNA expression, respectively (**Figures 2E, F**). Together, these results indicated that FBXW8 is an important member of E3 ligases on the suppression of PDCoV replication.

### 3.3 FBXW8 interacts with the PDCoV N protein dependent on its F-box domain

**Figure 3:**
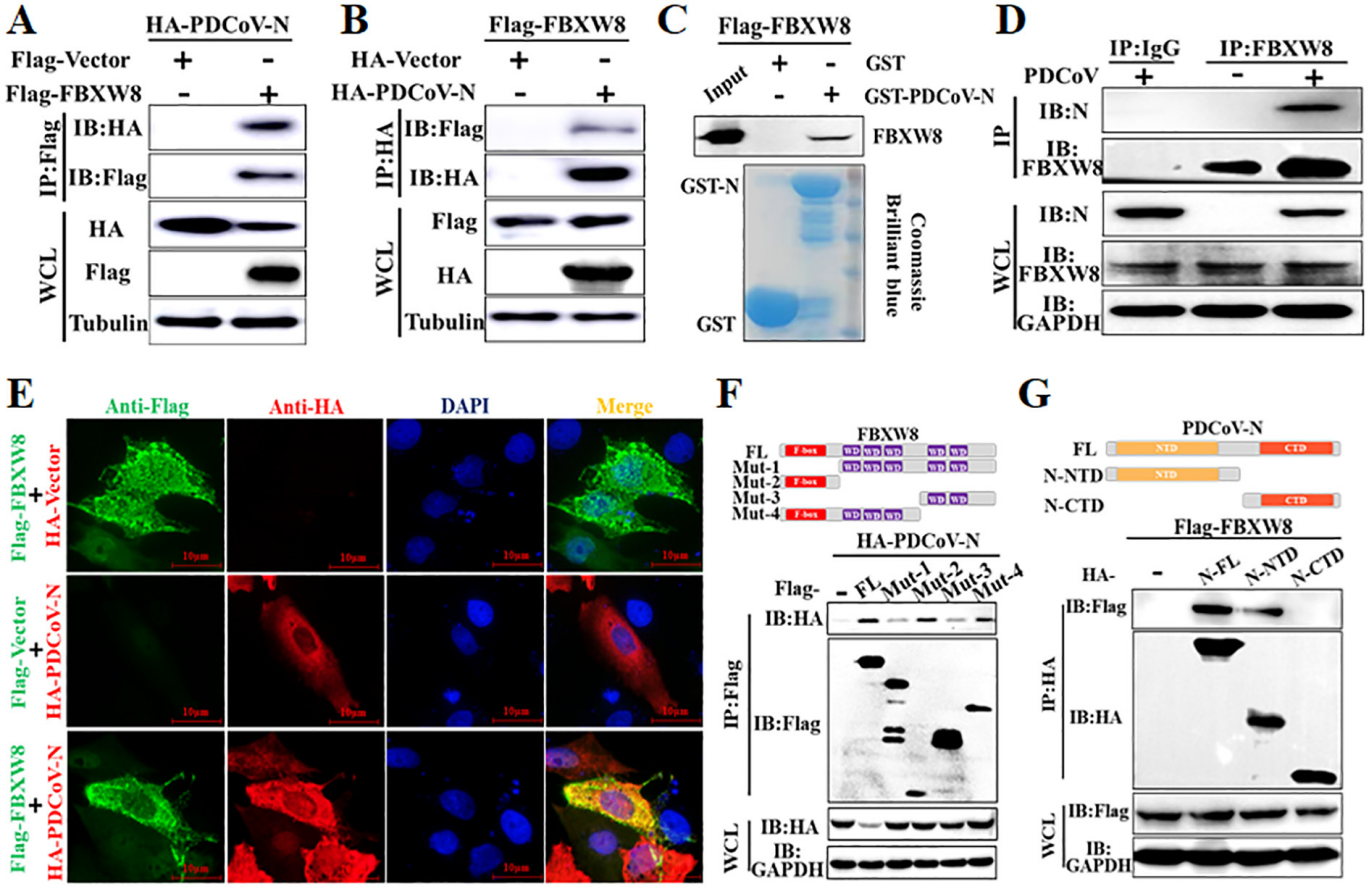
FBXW8 interacts with the PDCoV N protein. **(A, B)** HEK-293T cells were co-transfected with Flag-tagged FBXW8 and HA-tagged PDCoV N plasmids for 24 h, and then the whole cell lysate (WCL) was purified by anti-Flag **(A)** or anti-HA **(B)** affinity gel for immunoblot analysis, respectively. **(C)** PDCoV N were cloned into pET28a-GST vectors and transformed into BL21(DE3) cells. The relationship between FBXW8 and PDCoV N protein was assessed with the use of the GST pull-down kit. **(D)** LLC-PK1 cells were uninfected or infected with PDCoV (MOI = 0.1) for 28h. Co-IP assay was performed with the endogenous FBXW8 and couples to protein A/G beads. **(E)** PK-15 cells were transfected with FBXW8 and PDCoV N plasmids, which were labeled with specific primary antibodies (Flag is red and HA is green). Cell nuclei were stained with DAPI (blue). Confocal immunofluorescence microscopy was used to visualize the results. **(F)** Schematic representation of FBXW8 fragments used for Co-IP analysis (up). HEK-293T cells were co-transfected with HA-PDCoV-N and truncated fragments of Flag-FBXW8. **(G)** Schematic representation of PDCoV N protein used for Co-IP analysis (up). HEK-293T cells were co-transfected with Flag-FBXW8 and truncated fragments of PDCoV N protein. The WCL was performed for Co-IP assay with anti-Flag affinity gel. The WCLs and immunoprecipitants (IB) were analyzed by Western blot.

To determine the molecular mechanisms by which FBXW8 suppresses PDCoV replication, FBXW8 was identified as a potential interacting protein of the PDCoV N protein in our previous LC-MS/MS data (5). Hence, we firstly performed a coimmunoprecipitation (Co-IP) assay to verify the data. Co-IP results showed that the PDCoV N protein was precipitated by Flag-FBXW8 (**Figure 3A**), and Flag-FBXW8 was precipitated by PDCoV N (**Figure 3B**). Moreover, the glutathione S-transferase (GST) pulldown assay also verified the binding between FBXW8 and the PDCoV N protein. The prokaryotic expressed GST-fused PDCoV N protein (GST-PDCoV-N) obviously bound to FBXW8 but GST protein did not (**Figure 3C**), indicating that FBXW8 directly binds to the PDCoV N protein. Consistently, the result of IP assay showed that endogenous FBXW8 interacted with the PDCoV N protein in PDCoV-infected LLC-PK1 cells (**Figure 3D**). Furthermore, the IFA assay showed that the co-expressing FBXW8 (red) and PDCoV N protein (green) were colocalized in the cytoplasm by confocal microscopy (**Figure 3E**). The collective data demonstrated that FBXW8 directly interacted with the PDCoV N protein in the cytoplasm.

To investigate the interaction between FBXW8 and PDCoV N protein, four and two truncated mutants of FBXW8 (named Mut1–4) or PDCoV N protein (named N-CTD and N-NTD) were constructed depending on their conserved domains, respectively (**Figures 3F, G**). The Co-IP results showed that the truncated mutants containing F-box domain (Mut2 and Mut4) of FBXW8 were significantly coimmunoprecipitated with PDCoV N protein, which is consistent with the FBXW8 full-length (wt) (**Figure 3F**). However, the deletion F-box domain truncated mutants of FBXW8 (Mut1 and Mut3) hardly coprecipitated the PDCoV N protein (**Figure 3F**). Furthermore, Co-IP results showed that the N-terminal domain of PDCoV N (N-NTD), but not another truncated region (N-CTD), could clearly coprecipitate with FBXW8 (**Figure 3G**). Together, these results indicated that the FBXW8 depending on its F-box domain directly interacts with the N-terminal region of PDCoV N protein.

### 3.4 FBXW8 promotes PDCoV N protein polyubiquitination

**Figure 4:**
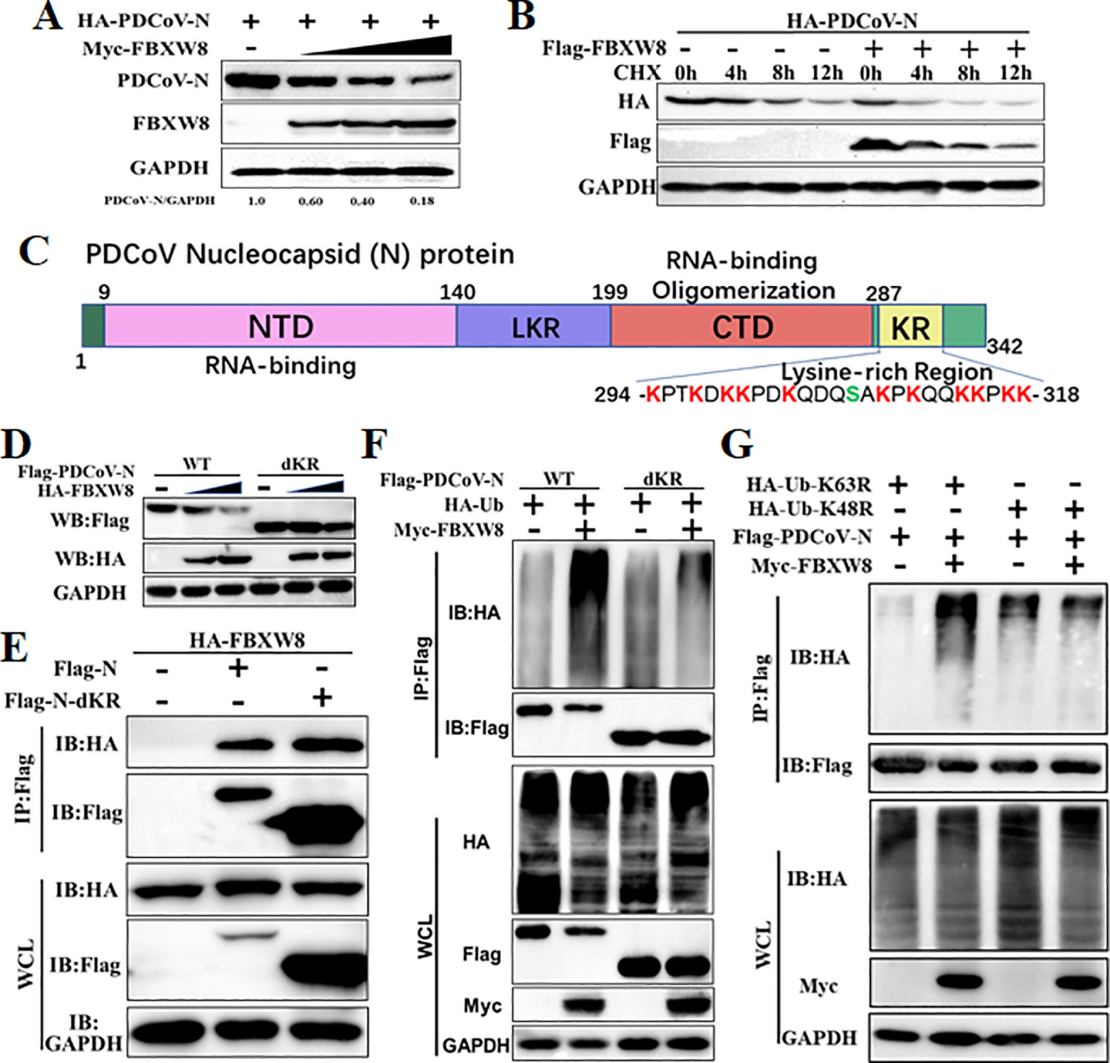
FBXW8 induced the polyubiquitination and degradation of PDCoV N protein. **(A)** PK-15 cells were transfected with HA-PDCoV-N (200 ng) with increasing amounts of Flag-FBXW8 plasmids (200, 400, or 800 ng) for 24h. **(B)** PK-15 cells were co-transfected with HA-PDCoV-N (300 ng) and Flag-FBXW8 (600 ng) or empty vector plasmids and then treated with protein synthesis inhibitor cycloheximide (CHX) 200 μg/mL for the indicated time before analysis of the protein levels by Western blot. **(C)** Schematic representation of PDCoV N protein conserved domains and motif. **(D)** PK-15 cells were co-transfected with Flag-PDCoV-N or KR-motif deletion mutant (Flag-PDCoV-NdKR) with increasing amounts of Flag-FBXW8 plasmid, and protein samples were collected for Western blot analysis. **(E)** HEK-293T cells were co-transfected with HA-FBXW8 and Flag-PDCoV-N or NdKR plasmids, **(F)** Flag-PDCoV-N or NdKR plasmids were co-transfected with Myc-FBXW8 or empty plasmids with HA-Ub, **(G)** HA-Ub-K48R or HA-Ub-K63R and Flag-PDCoV-N were co-transfected with Myc-FBXW8 or empty plasmids into HEK-293T cells, and then WCLs were purified by Flag-tagged affinity gel for immunoblot analysis.

To explore the mechanism of the FBXW8 target to the PDCoV N protein in inhibiting the virus replication, the PDCoV N protein was co-expressed with the empty vector or FBXW8 expression plasmids in PK-15 cells. Western blot analysis indicated that the PDCoV N protein was remarkably and dose-dependently decreased by FBXW8 (**Figure 4A**). It was consistent with the expression of the PDCoV N protein in PDCoV-infected cells (**Figure 2B**). Then, we performed cycloheximide (CHX) chase assay to analyze the half-life of the PDCoV N protein and found that FBXW8 increased the reduction rate of the PDCoV N protein (**Figure 4B**). These results indicate that FBXW8 interacts with the PDCoV N protein and then decreases its expression in cells.

Ubiquitination is an important PTM to regulate protein activation or degradation. Hence, we furthermore investigated whether the degradation of the PDCoV N protein induced by FBXW8 was due to ubiquitination. HEK-293T cells were co-transfected with Flag-tagged PDCoV-N, Myc-tagged FBXW8, or empty vector expression plasmids, together with the HA-tagged ubiquitin (Ub) plasmids for 24 h. The results revealed that FBXW8 significantly increased the polyubiquitination of the PDCoV N protein (**Figure 4F**). Furthermore, Co-IP results demonstrated that FBXW8 could not promote the polyubiquitination of the PDCoV N protein in Ub-K48R-expressed cells and significantly induced the polyubiquitination of the PDCoV N protein in Ub-K63R-expressed cells (**Figure 4G**). These findings indicated that FBXW8 could induce the K48-linked polyubiquitination of the PDCoV N protein. The PDCoV N protein revealed a unique conserved lysine-rich region (KR-motif) (**Figure 4D**). E3 ligase-mediated ubiquitination is usually achieved by adding Ub to the lysine residue of the substrate protein. Therefore, we explored the role of PDCoV N KR-motif in FBXW8-mediated ubiquitination. A KR-motif deletion mutant of the PDCoV N protein was constructed into the eukaryotic expression vector (named PDCoV-NdKR). Compared with the PDCoV N wild-type protein, the FBXW8-mediated degradation of the PDCoV N protein was significantly weakened (**Figure 4D**). However, Co-IP results showed that the KR-motif deletion did not affect the interaction between the PDCoV N protein and FBXW8 (**Figure 4E**). Moreover, FBXW8-induced ubiquitination of the PDCoV NdKR protein was also significantly reduced (**Figure 4F**). Taken together, all the results revealed that FBXW8 could bind to the PDCoV N protein, inducing its K48-linked polyubiquitination at KR-motif and degradation.

### 3.5 FBXW8 degrades the PDCoV N protein via NDP52-dependent selective autophagy

**Figure 5:**
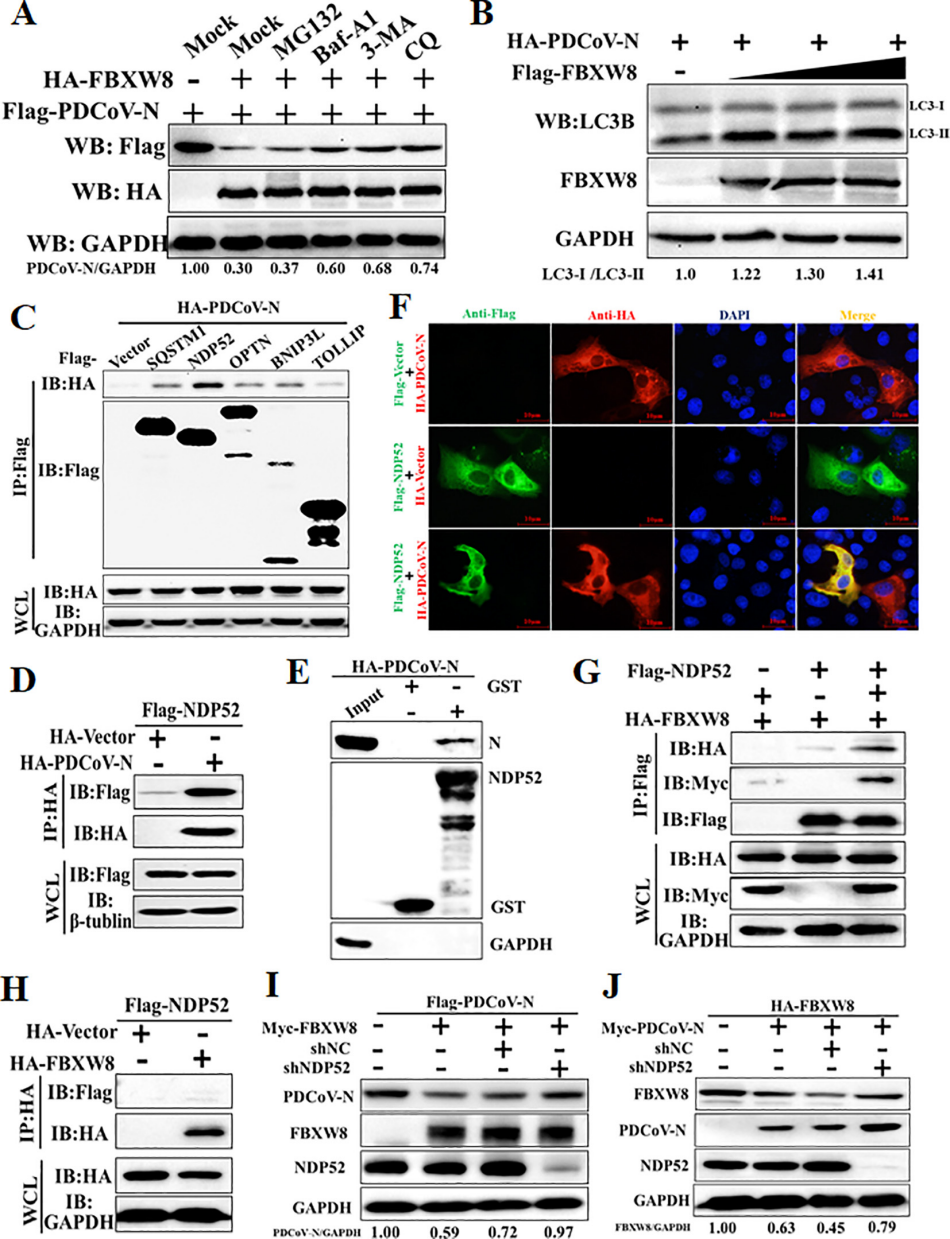
FBXW8 induces the autophagic degradation of PDCoV N protein via NDP52. **(A)** The FBXW8 and PDCoV N plasmid co-transfected cells were exposed to the treatment with ubiquitination inhibitor MG132, autophagy inhibitors 3-MA and CQ, and caspase inhibitor Z-VAD-FMK. **(B)** HEK-293T cells were transfected with HA-PDCoV-N and increasing concentrations of Flag-FBXW8 for 24h. The WCLs were analyzed with Western blotting. **(C)** The Flag-tagged cargo receptors (SQSTM1/p62, NDP52, OPTN, BNIP3L, and TOLLIP) were co-transfected with HA-PDCoV-N plasmids into HEK-293T cells, respectively. **(D)** HEK-293T cells were co-transfected Flag-NDP52 with HA-PDCoV-N plasmids, and the WCLs were purified by anti-Flag or anti-HA affinity gel for immunoblot analysis. **(E)** The pCold-GST-NDP52 plasmids were transformed into BL21(DE3) cells to induce protein expression. The relationship between NDP52 and PDCoV N protein was assessed with the use of the GST pull-down kit. **(F)** PK-15 cells were used for expression of Flag-NDP52 (green) and HA-PDCoV N protein (red). Cell nuclei were stained with DAPI (blue). Confocal immunofluorescence microscopy was used to visualize the results. **(G, H)** HA-FBXW8 and Flag-NDP52 together with Myc-PDCoV-N or empty plasmids were co-expressed in 293T cells, followed by a Co-IP assay utilizing Anti-Flag or Anti-HA affinity gels, respectively. **(I)** HEK-293T cells were co-transfected Flag-PDCoV N, Myc-FBXW8 with NDP52 shRNA or control (shNC). **(J)** HEK-293T cells were co-transfected Myc-PDCoV-N, HA-FBXW8 with NDP52 shRNA or shNC. The abundance of specific protein was analyzed by Western blot analysis.

The ubiquitinated protein is degraded mainly through the ubiquitin–proteasome system or the autophagy–lysosome pathway in eukaryotic cells (11). The PDCoV N and FBXW8 plasmids were co-transfected into HEK-293T cells to assess the FBXW8-induced N degradation pathways via treating with the proteasome inhibitor MG132 and the autophagy inhibitors 3-methyladenine (3MA) and chloroquine (CQ). Western blots showed that FBXW8-induced degradation of the PDCoV N protein was blocked by 3MA and CQ, but not by MG132 and Z-VAD-FMK (a caspase inhibitor) (**Figure 5A**). Moreover, co-expression of the PDCoV N protein with FBXW8 could increase the conversion of LC3-I to LC3-II (**Figure 5B**). Thus, these results indicated that FBXW8 modulated PDCoV N protein degradation through the autophagy–lysosome pathway.

The E3 ligase-mediated selective autophagy of substrate protein depends on cargo receptors, including SQSTM1/p62, CALCOCO2/NDP52, optineurin (OPTN), neighbor of BRCA1 (NBR1), BNIP3L, TOLLIP, and TAX1BP1 (26). In the present study, Co-IP assays were performed to examine which cargo receptors mediate the degradation of the PDCoV N protein after FBXW8-induced ubiquitination. Co-IP results showed that the PDCoV N protein was significantly precipitated by the NDP52 protein and slightly precipitated by SQSTM1/p62, OPTN, BNIP3L and TOLLIP (**Figures 5C, D**). The GST pulldown assay further validated the direct binding of the eukaryotic-expressed PDCoV N protein to NDP52 (**Figure 5E**). The IFA assay results also confirmed that the PDCoV N protein and NDP52 were colocalized in the cytoplasm (**Figure 5F**). Given that NDP52 serves as the primary cargo receptor of the PDCoV N protein, we concentrated on investigating the interaction between FBXW8 and NDP52. HA-tagged FBXW8 and Flag-tagged NDP52 plasmids were co-expressed in HEK-293T cells, followed by a Co-IP assay utilizing HA and Flag affinity gels, respectively. No obvious association between FBXW8 and NDP52 was found under basal conditions using Co-IP assay (**Figures 5G, H**). However, a notable formation of coprecipitates was observed in cells expressing Myc-PDCoV-N (**Figure 5I**). These results indicated that FBXW8-mediated PDCoV N protein polyubiquitination could recruit NDP52 forming protein complexes. To determine whether NDP52 is involved in FBXW8-induced autophagic degradation of the PDCoV N protein, PDCoV N and FBXW8, together with shNDP52 or negative-control shNC plasmids, were co-transfected into HEK-293T cells, respectively. At 24 h post-transfection, Western blotting was performed. We found that interfering with the expression of NDP52 effectively prevented the degradation of FBXW8-induced PDCoV N protein (**Figure 5I**). Moreover, silencing NDP52 could block the PDCoV N-induced degradation of FBXW8 protein (**Figure 5J**). Together, these results demonstrated that FBXW8 promoted the polyubiquitination of PDCoV N protein and autophagic degradation by forming an FBXW8-N-NDP52 complex.

## 4 Discussion

**Figure 6:**
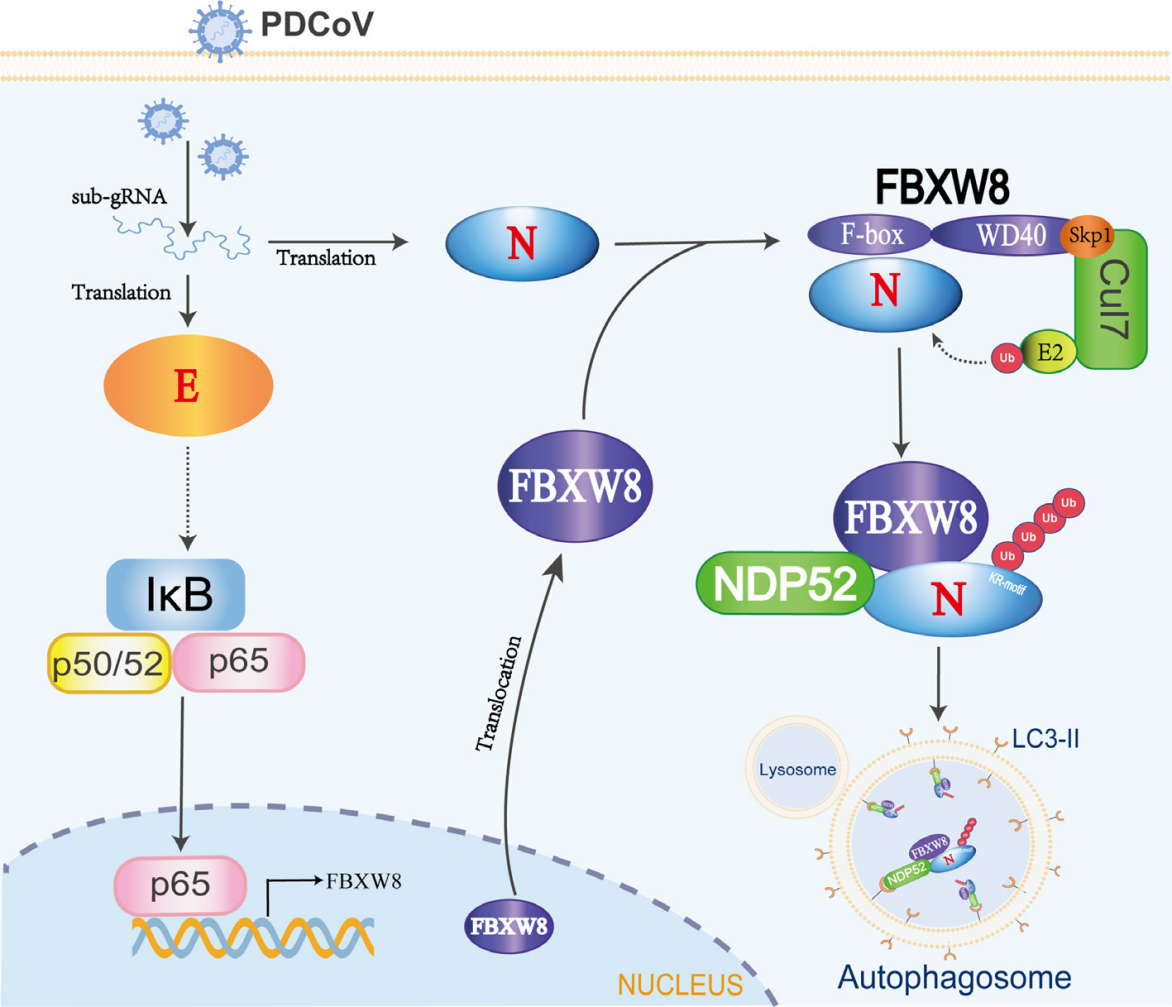
The mechanism of FBXW8 suppresses PDCoV proliferation. PDCoV releasing its genome or synthesis of a variety of viral proteins (such as E protein) could activate the NF-κB complex. The nuclear translocation of p65 directly increases FBXW8 expression by targeting its promoter. Moreover, PDCoV infection also induces FBXW8 translocation from nucleus to cytoplasm. In the cytoplasm, the F-box domain of FBXW8 directly interacts with PDCoV N protein to form a Cul7^FBXW8^ complex. The E2-carried ubiquitin molecules will be transferred to the lysine residue of PDCoV N KR-motif. The ubiquitinated PDCoV N protein is recognized by the cargo protein NDP52 and transported to autophagosomes for degradation.

PDCoV as an emerging pathogenic virus has caused huge economic losses on the pig industry. PDCoV escapes the host’s innate immune surveillance through a variety of strategies, benefiting its proliferation. However, the mechanisms of the host interfering with the replication of PDCoV are barely known. In the present study, we confirmed an underlying mechanism by which E3 ligase FBXW8 suppresses PDCoV replication in cells (**Figure 6**).

FBXW8-mediated degradation of substrate proteins regulates multiple cellular biological processes. Most research identified FBXW8 as part of the SCF^FBXW8^ complex to bind and induce the polyubiquitination of substrate, regulating cell cycle progression. There are limited reports on the impact of FBXW8 on virus replication, and this study hence focuses on investigating its antiviral function. We observed a significant increase in the FBXW8 protein during PDCoV infection at 36 h, while the FBXW8 mRNA level was decreased in PDCoV infection at 6 h (**Figures 1A, B**). PDCoV infection upregulated the expression of cytokines via the NF-κB signaling pathway in piglets and intestinal epithelial cells (27, 28). Furthermore, overexpression of the PDCoV E protein significantly activates the NF-κB complex, inducing the nuclear translocation of p65 (29). In the present study, p65 was upregulated in the later stage of PDCoV-infected PK-15 cells (**Figure 1B**). We identified p65 as the key transcription factor of FBXW8 to promote its transcription and upregulate its protein level in PK15 cells (**Figure 1E**). Overexpression of FBXW8 inhibited PDCoV replication, whereas downregulation of FBXW8 expression promoted virus proliferation (**Figure 2**). These findings suggested that PDCoV could downregulate FBXW8 transcription to benefit viral replication in the early infection step. However, PDCoV infection induced FBXW8 expression to suppress virus replication via activating the NF-κB signaling axis in the later infection stage (**Figure 6**).

An E3 ligase and viral protein forming a degradation complex, depending on specific host factors, is an important strategy of the host antiviral response. MARCH8, a host transmembrane protein E3 ligase, promotes the ubiquitination of SARS-CoV-2 S protein tail lysine and its subsequent lysosomal degradation (30). In the PEDV-infected cells, MARCH8 was recruited by several host antiviral proteins (BST2, HNRNPA1, FUBP3, HNRNPK, PTBP1, and TARDBP) to catalyze the polyubiquitination of the PEDV N protein and then promote the degradation of the N protein through the autophagy–lysosomal pathway (31). MARCH8 was also confirmed to indirectly target the PDCoV N protein via PABPC4 to induce its autophagic degradation (32). Furthermore, the E3 ligase STUB1 was recruited by PGAM5 to bind and degrade the PDCoV N protein via the autophagy–lysosomal pathway (33). Unlike MARCH8 and STUB1, FBXW8 was confirmed as a member of E3 ligases to directly interact with the PDCoV N protein in the present study (**Figures 3A–D**). Moreover, FBXW8 was co-located with the PDCoV N protein in the cytoplasm, but was not clear (**Figures 1C**, **3E**). FBXW8 induced the K48-linked polyubiquitination of PDCoV N at its KR-motif and subsequent degradation (**Figures 4**, **5A, B**). These findings suggest that PDCoV induced the relocation of FBXW8 in the cytoplasm, which facilitates the direct binding of the PDCoV N protein and the induction of N ubiquitination and degradation. The mechanism was consistent in that insulin induced FBXW8 cytoplasmic translocation to degrade IRS-1 (20). Furthermore, the unique KR-motif present in PDCoV N regulates its degradation and is not observed in the N proteins of other coronaviruses. Therefore, further investigation is warranted to determine if FBXW8 can induce degradation of N proteins from other coronaviruses.

Autophagy plays a dual role in virus infection and host antiviral responses. Coronaviruses could promote the formation of autophagosomes to provide basic structures for viral replication complexes. Moreover, the coronavirus enhances the breakdown of innate immune components in the host to diminish antiviral signals such as type I IFN and cytokines. PDCoV infection induced a complete autophagy process via the p38 signaling pathway to facilitate virus replication (34, 35). Pharmacologically inhibited autophagy with wortmannin and ergosterol peroxide (EP) suppresses the replication of PDCoV *in vitro* (34). Conversely, host cells can also suppress the replication of coronavirus through autophagy, particularly selective autophagy degrading the ubiquitinated viral components. There are seven cargo receptors that bind to ubiquitinated proteins, namely, SQSTM1/p62, CALCOCO2/NDP52, OPTN, NBR1, BNIP3L, TOLLIP, and TAX1BP1 (11). The PABPC4 and PGAM5 mediated the ubiquitinated PDCoV N protein via NDP52- and p62-dependent autophagic degradation, respectively (32, 33). In this study, FBXW8 has been confirmed to directly facilitate the polyubiquitination of the PDCoV N protein, which can be recognized and bound by a variety of autophagy PRRs, particularly NDP52 (**Figures 5C–E**). Silencing NDP52 expression significantly inhibited the degradation of the PDCoV N protein by FBXW8 (**Figure 5G**). Hence, these findings prove that FBXW8 is a novel member mediating the NDP52-dependent autophagic degradation. It is interesting to note that FBXW8 was also observed to simultaneously decrease with the PDCoV N protein in cells (**Figure 4B**). Moreover, shNDP52 could significantly block PDCoV N protein and FBXW8 expression in cells (**Figures 5I, J**). FBXW8 directly interacts with the N protein, while the N-dependent protein indirectly interacts with NDP52 (**Figures 4**, **5G**). All the above lines of evidence suggest that FBXW8, the PDCoV N protein, and NDP52 may form a protein degradation complex in cells (**Figure 6**). On one hand, increasing the expression and cytosolic transfer of FBXW8 is beneficial for the host against the proliferation of PDCoV by catalyzing its N protein autophagic degradation. On the other hand, the PDCoV N protein hijacks FBXW8 for synchronous degradation, thereby undermining the host’s antiviral response.

In conclusion, our study investigated the inhibitory effect of FBXW8 on PDCoV replication via the degradation of the PDCoV N protein. Mechanically, we have observed a correlation between FBXW8 and the PDCoV N protein in forming a protein complex, which is recognized by the cargo receptor NDP52 after FBXW8-mediated N ubiquitination, thereby promoting their delivery to autolysosome degradation.

## Data Availability

The original contributions presented in the study are included in the article/**Supplementary Material**. Further inquiries can be directed to the corresponding authors.
